# Sepsis in end-stage renal disease patients: are they at an increased risk of mortality?

**DOI:** 10.1080/07853890.2021.1987511

**Published:** 2021-10-09

**Authors:** Ralphe Bou Chebl, Hani Tamim, Gilbert Abou Dagher, Musharaf Sadat, Ghassan Ghamdi, Abdulrahman Itani, Alawi Saeedi, Yaseen M. Arabi

**Affiliations:** a Department of Emergency Medicine, American University of Beirut, Beirut, Lebanon; b Intensive Care Department, Ministry of National Guard Health Affairs, Riyadh, Kingdom of Saudi Arabia; c King Abdullah International Medical Research Center, Riyadh, Kingdom of Saudi Arabia; d King Saud Bin Abdulaziz University for Health Sciences, Riyadh, Kingdom of Saudi Arabia

**Keywords:** ESRD, sepsis, intensive care, mortality, critical care, lengths of stay

## Abstract

**Objectives:**

This study aims to examine the outcome of end-stage renal disease (ESRD) patients admitted with sepsis to the intensive care unit (ICU).

**Design:**

Single centre, retrospective cohort study

**Setting:**

The study was conducted in the Intensive Care Department of King Abdulaziz Medical City, Riyadh, Saudi Arabia.

**Participants:**

Data were extracted from a prospectively collected ICU database from 2002 to 2017. Patients were considered to have sepsis based on the sepsis-3 definition and were stratified into 2 groups based on the presence or absence of ESRD.

**Primary and secondary outcomes:**

The primary outcome of the study was in-hospital mortality. Secondary outcomes included ICU mortality, ICU and hospital lengths of stay, and mechanical ventilation duration.

**Results:**

A total of 8803 patients were admitted to the ICU with sepsis during the study period. 730 (8.3%) patients had ESRD. 49.04% of ESRD patients with sepsis died within their hospital stay vs. 31.78% of non-ESRD patients. ESRD septic patients had 1.44 greater odds of dying within their hospital stay as compared to septic non-ESRD patients (OR 1.44, 95% CI 1.03–1.53). Finally, the predictors of hospital mortality in septic ESRD patients were found to be mechanical ventilation (OR 3.36; 95% CI 2.27–5.00), a history of chronic liver disease (OR 2.26; 95% CI 1.26–4.07), and use of vasopressors (OR 1.74; 95% CI 1.19–2.54). Among patients with ESRD, hospital mortality was higher in subgroups of patients with chronic cardiac (OR 1.86 (1.36–2.53) vs. 1.19 (0.96–1.47)) and chronic respiratory illnesses (OR 2.20 (1.52–3.20) vs. 1.21 (0.99–1.48)).

**Conclusion:**

ESRD patients admitted to the intensive care unit with sepsis are at greater odds of mortality compared to patients with non-ESRD. This risk is particularly increased if these patients have a concomitant history of chronic cardiac and respiratory illnesses.

**Key Messages**
- Sepsis and bacterial infections are very common in ESRD patients and following cardiovascular disease; sepsis is the second leading cause of death in patients with ESRD.
- This study aims to examine the outcome of patients with end-stage renal disease (ESRD) patients admitted with sepsis to the intensive care unit (ICU).
- The results of this study have shown that end-stage renal disease is associated with greater odds of ICU and hospital mortality among septic patients admitted to an intensive care unit.
- ESRD patients were also more likely to be started on vasopressors and mechanical ventilation.

## Introduction

Sepsis is a life-threatening systemic inflammatory response to an infection that might result in organ injury, shock, or death [1,2]. In the United States, sepsis ranks as the 10th leading cause of death, and it accounts for 10% of all ICU admissions [3–6]. End-Stage Renal Disease (ESRD), defined as an irreversible decline in a person's kidney function, which is severe enough to be fatal in the absence of dialysis or transplantation, is a frequent comorbid factor in approximately 1 in 25 Emergency Department (ED) septic shock patients [7]. Moreover, an international surveillance investigation of hospitalised adults with septic shock identified 7.7% of patients to be on chronic dialysis [8]. Sepsis and bacterial infections are very common in ESRD patients and following cardiovascular disease; sepsis is the second leading cause of death in patients with ESRD [4,9–16]. Most sepsis studies have looked at the general population and have not looked at high-risk populations. Recently, two studies looked at the toll of sepsis on ESRD patients. A retrospective study done by Abou Dagher et al. found that the in-hospital mortality of septic haemodialysis patients (*n* = 90) was 26% and it was as high as 40% when they examined the subset of patients with septic shock [17]. Lowe et al. looked at the mortality of haemodialysis patients in septic shock (*n* = 137) and found that their ESRD patients had a mortality of 20.4% vs. 17% in non-ESRD patients [18]. However, both of these studies were done in the Emergency Department and they were limited by their small sample size. This study aims to look at haemodialysis (HD) patients admitted to the intensive care unit with sepsis and to compare their mortality and lengths of stay with non-ESRD patients.

## Methods

This is a single centre, retrospective, cohort study conducted in an academic ICU of King Abdulaziz Medical City, a large tertiary care centre in Riyadh, Saudi Arabia. The diagnosis of sepsis was constructed from the ICU database based on the sepsis-3 definition [19]. All adult patients (>18 years of age) who were admitted to the ICU between 2002 and 2017 and met the sepsis-3 criteria were included in the analysis. Patients who met the sepsis-3 definition were divided into two groups based on the presence of ESRD on ICU admission. For patients who were admitted more than once to the ICU within the same hospitalisation, we included the first admission only. Since we do not offer peritoneal dialysis or sustained low-efficiency dialysis (SLED) at our centre, these patients were not included in the study. Moreover, patients who developed acute kidney injury requiring renal replacement therapy as a complication of sepsis were excluded as this study aimed at looking at the toll of pre-existing ESRD on sepsis patients. Variables that were collected included the patients’ age, gender, acute physiology and chronic health evaluation (APACHE) II score, admission diagnosis category, vital signs at presentation, severe chronic comorbidities as defined by APACHE II system, history of cirrhosis, history of diabetes, Glasgow coma scale (GCS), mechanical ventilation requirement in the first 24 h of admission, the ratio of partial pressure of oxygen to the fraction of inspired oxygen (PaO2/FiO2), the requirement for vasopressors (defined as the use of any vasopressor infusion except dopamine <5 μ g/kg/min), admission bilirubin, creatinine, lactate, and international normalised ratio (INR), and ICU and hospital mortality, ICU and hospital length of stay (LOS), mechanical ventilation duration (MVD). APACHE II was calculated by a full-time data collector from the data collected at ICU admission and followed up to 24 h within the ICU. Multiple meetings were held between the PI, the research coordinator, and the research team to standardise their data collection. The primary outcome of the study is in-hospital mortality. Secondary outcomes included ICU and hospital lengths of stay, mechanical ventilation duration.

### Patient and public involvement

This is a retrospective chart review study where the patients were not involved in the study process. The study results will help guide our future management and identify the higher mortality risk of septic ESRD patients. The study was approved by the Institutional review board (IRB)- Ministry of National Guard Health Affairs.

### Statistical analysis

Statistical analysis software (SAS, version 9.0; SAS Institute, Cary, NC, USA) was used to analyse the data. Continuous variables were presented as median and interquartile ranges or mean and standard deviation as appropriate. Categorical variables were presented as frequencies and percentages. The chi-square and ANOVA tests were used to test significant differences between study groups. To determine the association between ESRD status and hospital mortality, bivariate and then multivariate logistic regression analyses were performed. The variables entered in the multivariable model were selected based on statistical as well as on clinical significance, and they included: Age; Gender; Admission category; Diabetes; Chronic cardiac disease; Chronic liver disease; Chronic respiratory disease; INR; APACHE II; lactic acid; vasopressor use; mechanical ventilation. ESRD patients were stratified according to survival to hospital discharge, and lengths of stays and mechanical ventilation duration of both cohorts were compared. Furthermore, another multivariable model, using the same clinically and statistically significant covariates, was done to test the effect modification of selected subgroups on the association between ESRD status and mortality. These subgroups included the following: male vs. female, age older than 50 years vs. age younger than 50 years, diabetes vs. no diabetes, mechanical ventilation vs. no mechanical ventilation, vasopressor use vs. no vasopressor use. Finally, a third multivariable analysis was done looking at only the ESRD cohort to find predictors of hospital mortality. The variables included in the model were Admission diagnosis; chronic liver disease; mechanical ventilation; INR; vasopressor use; APACHE II; Diabetes mellitus; Chronic cardiovascular disease; chronic respiratory disease; gender; age, lactic acid. Results were reported as odds ratio (OR) and 95% confidence interval (CI). A *P* value <.05 was considered statistically significant. Finally, immunosuppressed patients in our study were defined as patients receiving immunosuppressive agents, high dose steroids (e.g. methylprednisolone ≥15 mg/kg/day for ≥5 days), cancer patients on chemotherapy or radiotherapy, AIDS, and diffuse metastatic cancer patients.

## Results

### Patient characteristics

During the study period, 8803 patients met the sepsis-3 definition and were included in the study, of whom 730 (8.3%) were known to have end-stage renal disease. ESRD patients were older than non-ESRD patients (65.50 (±15.04) vs. 61.53 (±19.86).ESRD patients had a higher rate of chronic cardiac illness (32.42% vs. 26.02%, *p* < .0002), chronic liver disease (11.11% vs. 6.66%, *p* < .0001), and diabetes (63.01% vs. 46.92%, <0.001). Non-ESRD patients were more likely to be immunosuppressed (12.62% vs. 5.08%, *p* < .0001). Moreover, ESRD patients had a higher creatinine level (415.1 µmol/l (±217.4) vs. 140.4 µmol/l (±126.5), *p* < .001) and APACHE II scores (28.69 vs. 22.99, *p* < .0001). There was no statistically significant difference in lactic acid levels between the two groups. These results are summarised in Table 1.

**Table 1.** Demographics, baseline characteristics, laboratory values of ESRD and non-ESRD patients admitted to the ICU with sepsis.

Variable	Non-ESRD	ESRD	*p*-value
	*N* = 8073	*N* = 730	
Female gender, *N* (%)	3603 (44.63)	349 (47.81)	.098
Age (year), mean ± SD	61.53 ± 19.86	65.50 ± 15.04	<.0001
Admission diagnosis, *N* (%)			
Non-operative	7557 (93.61)	707 (96.85)	.0005
Post-operative	516 (6.39)	23 (3.15)	
Chronic diseases, *N* (%)			
Cardiac disease	2096 (26.02)	236 (32.42)	.0002
Respiratory disease	2211 (27.46)	169 (23.21)	.0137
Liver disease	536 (6.66)	81 (11.11)	<.0001
Immunosuppression	1016 (12.62)	37 (5.08)	<.0001
Tracheostomy, *N* (%)	807 (10.0)	78 (10.68)	.5535
Vasopressor, *N* (%)	3429 (42.47)	426 (58.36)	<.0001
Diabetes Mellitus, *N* (%)	3788 (46.92)	460 (63.01)	<.0001
Mechanical ventilation, *N* (%)	4869 (60.31)	469 (64.25)	.0372
APACHE II, mean ± SD	22.99 ± 8.11	28.69 ± 7.78	<.0001
Bilirubin, µmol/l, mean ± SD	36.83 ± 80.68	39.55 ± 88.50	.4688
GCS, mean ± SD	11.46 ± 3.96	10.91 ± 4.04	.0004
Creatinine, µmol/l, mean ± SD	140.4 ± 126.5	415.1 ± 217.4	<.0001
Lactic Acid, mg/dl, mean ± SD	3.10 ± 3.43	3.53 ± 3.95	.0076
INR, mean ± SD	1.56 ± 1.05	1.69 ± 1.07	.0022
PaO2/FiO2, mean ± SD	204.6 ± 117.6	219.4 ± 129.1	.0033

APACHE II: Acute Physiology and Chronic Health Evaluation II; GCS: Glasgow Coma Scale; INR: International normalised ratio; PaO_2_/FiO_2:_ the ratio of partial pressure of oxygen to the fraction of inspired oxygen.

### ICU management and mortality

**Figure 1.**
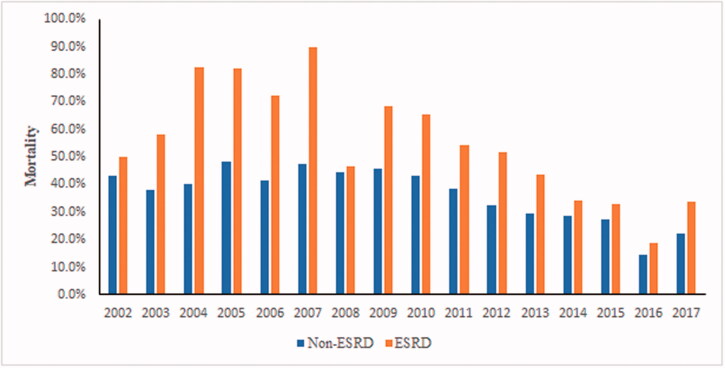
Temporal distribution of ESRD and Non-ESRD mortalities between 2002 and 2017.

ESRD patients had higher hospital mortality than non-ESRD (49.04% vs. 31.78%, *p* < .0001), as well as higher ICU mortality (29.52% vs. 18.50%, *p* < .0001) (Table 2). There was also a decreasing trend in mortality in both cohorts between 2004 and 2017 (Figure 1). Furthermore, ESRD patients were more likely to get intubated than non-ESRD (64.25% vs. 60.31%, *p* .037), and after stratifying by survivors, ESRD patients had a longer mechanical ventilation duration (median 3.00 IQR 10.00 vs. median 2.00 IQR 8.00; *p* .028). A higher percentage of ESRD patients (58.36% vs. 42.47%; *p* < .0001) required vasopressors during their hospital stay. On multivariable analysis, ESRD was found to have greater odds of in-hospital mortality (OR 1.44, 95% CI 1.21–1.72), as well as ICU mortality (OR 1.26, 95% CI 1.03–1.53) while adjusting for other confounders (Table 3).

**Table 2.** Outcome differences between ESRD and non-ESRD cohorts.

	No ESRD	ESRD	*p*-value
	*N* = 8073	*N* = 730	
ICU mortality, *N* (%)	1472 (18.50)	214 (29.52)	<.0001
Hospital Mortality, *N* (%)	2560 (31.78)	358 (49.04)	<.0001
ICU Length of stay (LOS) (Median, IQR)	4.25 (8.95)	6.19 (11.60)	.0151
Hospital length of stay (LOS) (Median, IQR)	23.00 (38.00)	28.00 (42.00)	.0051
Mechanical ventilation duration, (Median, IQR)	2.00 (8.00)	3.00 (10.00)	.0278

ICU: Intensive care unit; LOS: length of stay; *ICU mortality missing for 120 patients ESRD: 5, Non-ESRD: 115; *Hospital mortality missing for 17 patients Non-ESRD: 17.

**Table 3.** Multivariate analysis for primary and secondary outcomes between the ESRD and non-ESRD cohorts.

Categorical outcomes						
	No ESRD	ESRD	*p*-value	OR ( 95 % CI )	*p*-value	
	*N* = 8073	*N* = 730				
ICU mortality, *N* (%)	1472 (18.50)	214 (29.52)	<.0001	1.26 (1.03 ; 1.53)	.02	
Hospital mortality, N (%)	2560 (31.78)	358 (49.04)	<.0001	1.44 (1.21 ; 1.72)	<.0001	
Continuous outcomes among hospital survivors						
ICU length of stay, (LOS) (Median, IQR)	4.25 (8.95)	6.19 (11.60)	.015	2.04 (0.31 ; 3.76)	.02	
Hospital length of stay, (LOS) (Median, IQR)	23.00 (38.00)	28.00 (42.00)	.005	10.08 (3.87 ; 16.28)	.002	
Mechanical ventilation duration, (Median, IQR)	2.00 (8.00)	3.00 (10.00)	.03	6.98 (0.41; 13.55)	.037	

The following variables were included in the model; Age; Gender; Admission category; Diabetes; Chronic cardiac disease; Chronic liver disease; Chronic respiratory disease; International normalised ratio(INR); Acute Physiology and Chronic Health Evaluation II(APACHE II); lactic acid; vasopressor use; mechanical ventilation.

### Mortality predictors

The most important predictors of hospital mortality in septic ESRD patients were found to be mechanical ventilation (OR 3.36; 95% CI 2.27–5.00), and a history of liver disease (OR 2.26; 95% CI 1.26–4.07), and use of vasopressors (OR 1.74; 95% CI 1.19–2.54). The predictors of mortality are all summarised in Table 4.

**Table 4.** Predictors of hospital mortality among ESRD patients admitted to the ICU with sepsis.

Parameters	Odds ratio (OR)	95% confidence interval (CI)
Age (per 1-year increase)	1.01	1.00–1.02
Sex	1.17	0.82–1.66
APACHE II (per 10-unit increase)	2.46	1.80–3.35
INR (per 1-unit increase)	1.14	0.94–1.39
Admission Diagnosis	0.96	0.81–1.15
Mechanical ventilation	3.36	2.27–5.00
Lactic Acid (>2mmol/L)	1.21	0.83–1.77
Vasopressors	1.74	1.19–2.54
Diabetes mellitus	0.88	0.61–1.28
Chronic cardiovascular	1.31	0.90–1.92
Chronic respiratory illness	1.32	0.85–2.04
Chronic liver disease	2.26	1.26–4.07

The following variables were included in the model: Admission diagnosis; chronic liver disease; mechanical ventilation; International normalised ratio (INR); vasopressor use; Acute Physiology and Chronic Health Evaluation II (APACHE II); Diabetes mellitus; Chronic cardiovascular disease; chronic respiratory disease; gender; age, lactic acid.

### Subgroups analysis

The effect modification of the association between ESRD and hospital mortality was higher in patients with chronic cardiac (OR 1.86 (1.36–2.53) vs. 1.19 (0.96–1.47), *p* value for interaction .02) and chronic respiratory illnesses (OR 2.20 (1.52–3.20) vs. 1.21 (0.99–1.48) *p* value for interaction .002) (Table 5). There was no heterogeneity in the association of ESRD and mortality in the other subgroups.

**Table 5.** Multivariate subgroup analysis by different baseline characteristics for the association between ESRD status and hospital mortality.

	No ESRD	ESRD	OR (95 % CI )	*p*-value	Tests of interaction
	*N* = 8073	*N* = 730			
Gender					
Male	1461 (32.74)	182 (47.77)	1.29 (1.01–1.65)	.04	0.56
Female	1099 (30.58)	176 (50.43)	1.61 (1.25–2.08)	.0002	
Age					
<50	414 (20.52)	38 (37.25)	1.39 (0.85–2.31)	.19	0.67
>50	2146 (35.54)	320 (50.96)	1.42 (1.17–1.71)	.0003	
Diabetes					
No	1342 (31.36)	128 (47.41)	1.25 (0.94–1.67)	.0015	0.49
Yes	1218 (32.25)	230 (50.00)	1.52 (1.22–1.89)	.0002	
Mechanical ventilation					
No	510 (15.96)	57 (21.84)	1.18 (0.86–1.63)	.30	0.16
Yes	2050 (42.17)	301 (64.18)	1.52 (1.23–1.89)	<.0001	
Non-operative diagnosis					
No	2470 (32.76)	347 (49.08)	1.39 (1.16–1.66)	.0003	0.46
Yes	90 (17.44)	11 (47.83)	2.18 (0.79–5.99)	.13	
Vasopressors					
No	1023 (22.06)	95 (31.25)	1.19 (0.91–1.57)	.21	0.12
Yes	1537 (44.95)	263 (61.74)	1.58 (1.26–2.00)	.0001	
Chronic cardiac disease					
No	1854 (31.08)	225 (45.55)	1.19 (0.96–1.47)	.12	0.02
Yes	706 (33.76)	133 (56.36)	1.86 (1.36–2.53)	<.0001	
Chronic respiratory disease					
No	1947 (33.27)	260 (46.35)	1.21 (0.99–1.48)	.07	0.002
Yes	613 (27.81)	98 (57.99)	2.20 (1.52–3.20)	<.0001	
Lactic acid					
<2mmol/L	1268 (25.57)	169 (40.33)	1.43 (1.14–1.80)	.0023	0.93
>2mmol/L	1292 (41.72)	189 (60.77)	1.39 (1.06–1.83)	.02	

The following variables were included in the model; Age; Gender; Admission category; Diabetes; Chronic cardiac disease; Chronic liver disease; Chronic respiratory disease; International normalised ratio(INR); Acute Physiology and Chronic Health Evaluation II(APACHE II); lactic acid; vasopressor use; mechanical ventilation.

## Discussion

The results of this study have shown that end-stage renal disease is associated with greater odds of ICU and hospital mortality among septic patients admitted to an intensive care unit. ESRD patients were also more likely to be started on vasopressors and mechanical ventilation.

These results are in agreement with a study done by Sarnak et al. (*n* = 2746) which showed that mortality due to sepsis was 50 folds higher in haemodialysis patients as compared to the general population [13]. In another study by Sakhuja et al. (*n* = 322,734) the authors found that mortality from severe sepsis in patients on maintenance dialysis was higher than the general population (30.3% vs. 26.2%; *p* < .001) [20].

Our results have also shown that the mortality of septic haemodialysis patients remains much higher (47.65%) than the sepsis-related mortality in the general population [21–23]. This increased mortality can be explained by the higher predisposition to bacterial infections in ESRD patients as opposed to non-ESRD patients [10,11,24]. This increased susceptibility to bacterial infections is due in part to underlying immune dysfunctions. Defective phagocytic function of granulocytes, impaired monocyte function, as well as impaired T lymphocyte maturation has been described in patients on chronic dialysis [25–30]. The immunocompromised state of uraemia, age, and diabetes, as well as the frequent use of intravascular catheters in haemodialysis patients, have been associated with increased rates of infections. Finally, due to impaired renal function, ESRD patients are at risk of inflammatory cytokine accumulation, which can further attenuate their immune function [31–34]. It is important to note that even though ESRD patients have higher mortality than non-ESRD, there was a decreasing trend in sepsis-related mortality in both cohorts between 2004 and 2017. This is in line with the literature that shows an overall decrease in sepsis-related mortality after several studies reinforced early recognition and early antibiotics in sepsis care [21,35].

In our cohort, septic haemodialysis patients were more likely to be started on vasopressors (54.58% vs. 42.31%, *p* < .001) than the non-ESRD group. They were also more likely to be intubated than the non-ESRD group. Oppert et al. noted in their observational study (*n* = 166) that septic patients with renal failure required more frequent use of vasopressor therapy compared with patients without renal failure [2,36]. This is probably because it is clinically challenging to determine dialysis patients’ intravascular volume status and fluid responsiveness [18]. Moreover, physicians may be reluctant to give IV fluid resuscitation to ESRD patients as they assume volume overload in dialysis-dependent patients and may be more inclined to start these patients on vasopressors. According to Lowe *et.al* (*n* = 137), ESRD septic shock patients received less fluid resuscitation (11 mL/kg in the first 3 h) compared with non-ESRD septic shock patients despite having similar presenting physiologic variables and shock markers [18].

Finally, according to Sarnak and Lowe, Age, DM and hyperlactatemia were associated with a higher rate of death in ESRD septic patients [13,18]. Our results have shown that the factors associated with an increased ICU and hospital mortality were a co-existing history of liver disease, mechanical ventilation, incremental increase in the APACHE II score, and the use of vasopressors. This is in line with the literature that shows that mechanical ventilation is associated with poor outcomes in critically ill patients [37,38]. Furthermore, when we looked at the effect modifications of certain subgroups on the interaction of ESRD and mortality, we observed that mortality is increased in ESRD patients with concomitant chronic respiratory and cardiac illnesses. As such, physicians should be aware of this high-risk population and be cognisant of the factors that increase their mortality.

### Limitations

This was a retrospective study and as such, the authors are aware of the inherent limitations of such a type of study. However, we believe that this was overcome by the large sample size of the study. Some of the limitations of this retrospective database are the lack of important variables such as the cause of ESRD, time to antibiotics, time to diagnosis, type of vascular access, and volume of resuscitation. This study was done at a referral centre that deals with challenging cases and referrals. As such, our results might not be generalisable to other institutions. Finally, although ESRD patients were more likely to get started on vasopressors, and this was found to be a predictor of mortality. This was not one of the primary endpoints of this study, and as such, we cannot draw any conclusions regarding the effect of vasopressors in the treatment of septic ESRD patients.

## Conclusion

Septic patients admitted to the intensive care unit with a known history of ESRD have increased hospital mortality. The mortality effect is even higher in patients who have chronic respiratory and cardiac illnesses. Future studies are needed to look at interventions and management strategies tailored specifically for this high-risk population.
